# The Clinical, Functional, and Radiological Outcomes of Percutaneous Laser Disc Decompression in Disc-Related Lumbar Spinal Stenosis: A Retrospective Cohort Study

**DOI:** 10.3390/jcm15114060

**Published:** 2026-05-24

**Authors:** Cagatay Kucukbingoz, Ahmet Yilmaz

**Affiliations:** Ministry of Health, Adana City Training & Research Hospital, Adana 01370, Turkey; dr.ahmetyilmaz27@gmail.com

**Keywords:** spinal stenosis, percutaneous laser disc decompression, radicular pain, MCID, functional improvement, indirect decompression

## Abstract

**Objective:** This study aimed to evaluate the clinical and radiological efficacy of percutaneous laser disc decompression (PLDD) in patients with disc-related lumbar spinal stenosis. **Methods:** Data from 96 patients who underwent PLDD between January 2023 and January 2025 were reviewed retrospectively. Pain intensity (visual analogue scale [VAS]), functional capacity (pain-free walking distance), patient satisfaction (global patient evaluation), and radiological canal diameter were assessed before the procedure and at 1, 3, and 6 months postoperatively. Treatment response was determined based on a ≥2-point decrease in the VAS score, which is the minimal clinically important difference (MCID) criterion. **Results:** A marked improvement in VAS scores was observed from the early period following PLDD, with the mean VAS score decreasing from 8.02 to 5.02 ± 1.99 at 6 months (*p* < 0.001). The pain-free walking distance increased from 212.7 m to 345.8 m, resulting in a significant improvement in functional capacity (*p* < 0.001). A significant increase in the anteroposterior diameter of the spinal canal from 7.1 ± 1.7 mm to 7.9 ± 1.8 mm (*p* < 0.001) was observed, corresponding to a mean increase of 0.8 mm; however, the magnitude of this radiological change was modest and should be interpreted cautiously. A moderate correlation was found between radiological expansion and VAS change (r = 0.52). At 6 months, 72.9% of patients met the MCID criterion. Although ODI improved significantly over follow-up, the mean reduction remained below commonly accepted MCID thresholds, suggesting that the functional benefit may be modest. No major complications were observed; only short-term transient radicular irritation (2.1%) was seen. **Conclusions:** PLDD was associated with improvements in pain control, functional capacity, and modest radiological canal enlargement in this cohort of carefully selected patients with single-level, predominantly disc-driven lumbar spinal stenosis. However, because of the retrospective design and absence of a control group, no conclusions regarding comparative effectiveness can be drawn. PLDD should therefore be viewed as a selectively applicable minimally invasive option rather than a general treatment for all forms of lumbar spinal stenosis. The observed clinical benefit was limited to the 6-month follow-up available in this cohort, and its durability beyond this period remains uncertain. Prospective and comparative studies are required to better define its long-term role and its position relative to conservative treatment and surgery.

## 1. Introduction

Lumbar spinal stenosis is a common cause of radicular pain and functional limitation in adults. In a subset of patients, stenotic narrowing is predominantly related to disc pathology, including contained disc herniation, protrusion, or extrusion, which may contribute to canal, lateral recess, or foraminal compromise. Its pathophysiology involves increased nucleus pulposus pressure, annulus fibrosus weakening, and mechanical or chemical irritation of the nerve roots resulting from degeneration [1,2]. Minimally invasive techniques are becoming more important as potential treatment options in the stepwise management of patients who do not respond adequately to conservative treatment, rather than as definitive alternatives to surgery [3].

First introduced into clinical practice by Choy, percutaneous laser disc decompression (PLDD) is based on the principle of reducing intradiscal pressure using laser energy [4]. Experimental studies have shown that laser energy reduces intradiscal pressure by providing controlled vaporization within the nucleus and contributes to the retraction of the herniated fragment [5,6]. It has also been suggested that an increase in intradiscal temperature may reduce the sensitivity of nerve fibres [6].

Numerous studies have evaluated the clinical efficacy of PLDD. A series of 71 patients from Turkey reported significant pain reduction [7], while a prospective study of 30 patients demonstrated marked improvements in both visual analogue scale (VAS) and functional scores [8]. Gazzeri et al. demonstrated that PLDD significantly improved both pain and functional capacity in patients with contained disc herniation [9]. A comparative study with a larger sample size reported that PLDD was significantly superior to conservative treatment [10]; however, such comparative findings should be interpreted cautiously, as the overall evidence remains limited and heterogeneous.

When evaluated radiologically, long-term follow-up studies have reported a modest but significant reduction in disc volume and canal diameter after PLDD. A 2-year study by Hashemi et al. demonstrated a correlation between the significant regression of disc protrusion and clinical improvement [11]. However, the magnitude and clinical relevance of radiological changes remain variable across studies.

Guidelines state that intradiscal minimally invasive techniques are rational options for patients who are not candidates for surgery and do not respond adequately to conservative treatments [3]. In this context, PLDD has been proposed as a technique with low invasiveness and complication rates and short recovery times in selected patient populations.

Lumbar spinal stenosis presents a heterogeneous spectrum in terms of pathology, radiology, and clinical manifestations; therefore, demographic and radiological factors, such as age, sex, canal area, lateral recess narrowing, type of disc pathology, and accompanying degenerative changes, reportedly influence the clinical response following PLDD. The evaluation of these variables is important for appropriate patient selection and the prediction of treatment success [9,10,11]; however, these associations have not been consistently confirmed across studies.

In practical terms, PLDD should be considered primarily for carefully selected patients with single-level, predominantly disc-driven lumbar spinal stenosis, particularly when symptoms persist despite conservative treatment and when a minimally invasive intermediate step is preferred before surgery. In contrast, patients with marked segmental instability, advanced spondylolisthesis, cauda equina findings, prior lumbar surgery, or stenosis predominantly driven by non-discal multilevel degenerative changes should not be regarded as ideal candidates for this procedure.

This study aimed to comprehensively analyze the demographic, clinical, and radiological characteristics of 96 patients with spinal stenosis who underwent PLDD. The main objectives of this study were to (i) demonstrate the clinical outcomes associated with PLDD in lumbar spinal stenosis, (ii) evaluate the relationship between radiological parameters and clinical response, and (iii) identify predictive factors that could optimize patient selection within the limitations of a retrospective, single-centre design without a control group.

## 2. Materials and Methods

### 2.1. Study Design and Ethical Approval

This single-centre, retrospective cohort study analyzed data from patients who underwent PLDD at our clinic between January 2023 and January 2025 and was not designed to compare PLDD with other treatment modalities. This study was conducted in accordance with the 2013 revision of the Declaration of Helsinki and approved by the relevant local ethics committee (Decision No: 582/2025; Date: 10 July 2025). All patients were provided with detailed information about the procedure, potential risks, and follow-up process before the procedure, and their written informed consent was obtained.

### 2.2. Patient Selection

Cases were included in this study according to clinical, functional, and radiological criteria. Patient age, symptom duration, comorbidities, magnetic resonance imaging (MRI) findings, and responses to conservative treatment were recorded during evaluation to ensure appropriate patient characterization within the limitations of a retrospective study design.

The inclusion criteria were as follows:

- Age range of 18–85 years.
- MRI-confirmed disc-related lumbar spinal stenosis at a single level, characterized by posterolateral disc protrusion or extrusion contributing to canal, lateral recess, or foraminal narrowing.
- Symptoms persisted despite at least 6 weeks of conservative treatment.
- Preoperative VAS score ≥ 5.
- Clinically significant limitations in pain-free walking distance.

The exclusion criteria were as follows:

- Spondylolisthesis grade > I.
- History of lumbar surgery.
- Modic type III vertebral changes.
- Structural spinal pathologies such as infection, discitis, tumour, and fracture.
- Findings suggestive of cauda equina syndrome.

In routine practice, PLDD is considered most suitable for patients with persistent radicular pain and/or neurogenic walking limitation attributable predominantly to a contained or mildly extruded disc component at a single lumbar level, after failure of conservative treatment. The procedure is not considered an ideal option for patients with major instability, high-grade spondylolisthesis, cauda equina syndrome, prior lumbar surgery, or structural compression patterns unlikely to respond to indirect intradiscal decompression.

In addition, comorbidities such as body mass index (BMI), smoking, diabetes, hypertension, and osteoporosis were systematically recorded in all patients and evaluated as potential confounding variables in the statistical analyses. Symptom duration was recorded in months and analyzed in terms of two categories, ≤6 months and >6 months, as potential covariates in outcome analyses.

### 2.3. Clinical Assessment

Patients underwent clinical assessments before the procedure and at 1, 3, and 6 months post-procedure. The following clinical outcomes were assessed at each time point:

- Pain level: This was measured using the Turkish version of the validated and reliable VAS (score range, 0–10) [12].
- Disability level: Patients’ functional limitations and the extent to which daily living activities were affected were assessed using the Turkish version of the validated and reliable Oswestry Disability Index (ODI). The ODI is scored on a scale of 0–100, with higher scores indicating greater disability, and was interpreted with reference to the minimal clinically important difference (MCID) thresholds reported in the literature.
- Functional capacity: This was assessed using the 6 min walk test (6MWT), which was administered in accordance with the American Thoracic Society guidelines. The clinical conditions were kept constant, and the test was performed by the same trained nurse using standard commands.
- Patient satisfaction: This was assessed using the global patient evaluation (GPE) scale with a 1–7-point rating.
- Minimal clinically important difference (MCID) definition: A ≥2-point decrease in the VAS score and/or ≥50% improvement from baseline was considered a “responder” for clinical significance for primary outcome interpretation.

### 2.4. Radiological Evaluation

All patients underwent lumbar MRI before the procedure and at 3 months post-procedure. Images were obtained using systems with similar technical specifications and a magnetic field strength of 1.5 Tesla (Device/Model: Philips Ingenia, Philips Healthcare, Best, The Netherlands; Siemens Avanto, Siemens Healthineers, Erlangen, Germany). The MRI protocol and evaluated parameters included the following:

- Sagittal and axial T2-weighted sequences;
- Slice thickness of 3 mm;
- Anteroposterior canal diameter measurements;
- The anteroposterior dimensions of herniation;
- The degree of nerve root compression.

All MRI measurements were performed independently by two investigators who were blinded to the clinical outcomes and treatment allocation. Inter-measurement agreement was assessed using the intraclass correlation coefficient (ICC), which was >0.85, thus indicating high measurement reliability, although small absolute changes should be interpreted with caution.

### 2.5. PLDD Procedural Technique

**Figure 1:**
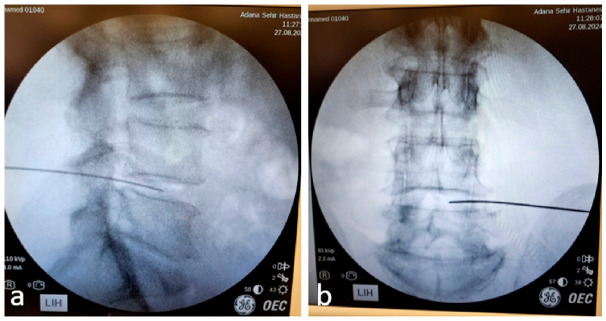
Fluoroscopic guidance during percutaneous laser disc decompression (PLDD). (**a**) Lateral fluoroscopic view showing advancement of the spinal needle into the target intervertebral disc space. (**b**) Anteroposterior fluoroscopic view confirming appropriate needle position before laser application.

All procedures were performed by the same experienced interventional pain specialist. Patients were positioned prone; 2 cc of 1% lidocaine was administered as local anesthesia, and minimal sedation with low-dose midazolam was provided when necessary. The procedural steps and technical parameters were as follows:

- Posterolateral transforaminal approach;
- Advancement of a 20G spinal needle into the disc;
- Verification of the needle position using anteroposterior and lateral fluoroscopy (Figure 1);
- Use of a 980 nm wavelength diode laser;
- Application of a total of 1500 Joules of energy in the pulsed mode.

The aim was to reduce intradiscal pressure and decrease the mechanical load on the nerve root by creating controlled vaporization in the nucleus pulposus; however, the extent of structural change achievable with this energy level is limited and should not be interpreted as a purely mechanical decompression effect. The average fluoroscopy time was 11–18 s, and all procedures were completed with low radiation exposure. Postoperatively, patients received oral amoxicillin–clavulanic acid (875 mg/125 mg) twice daily for 10 days for prophylaxis. Additionally, a 3-day course of a nonsteroidal anti-inflammatory drug, 1 week avoiding heavy lifting, and a 2-week controlled physical therapy program were recommended.

### 2.6. Endpoints

The primary endpoints were the change in the VAS score at 6 months (ΔVAS) and the MCID responder rate. The secondary endpoints were the change in pain-free walking distance, change in radiological canal diameter, degree of nerve root compression, GPE satisfaction scores, achievement of a ≥50% improvement rate, and incidence of complications without comparative evaluation against alternative treatment modalities.

### 2.7. Statistical Analysis

Continuous variables were tested for normality using the Shapiro–Wilk test and are presented as the mean ± SD or median (IQR), as appropriate; categorical variables are reported as n (%). Changes in outcomes over follow-up were analyzed using a repeated-measures ANOVA for normally distributed data and the Friedman test otherwise, with Bonferroni adjustment for post hoc multiple comparisons. Effect sizes were calculated using Cohen’s d. Clinical response was defined using a prespecified MCID threshold, classifying patients as responders or non-responders at the primary follow-up time point. Independent predictors of MCID response were evaluated using multivariable logistic regression, and the results are reported as odds ratios with 95% confidence intervals. A two-sided *p*-value < 0.05 was considered statistically significant. Statistical analyses were performed using IBM SPSS Statistics for Windows, Version 28.0 (IBM Corp., Armonk, NY, USA). No adjustments were performed for multiple subgroup comparisons, and subgroup analyses should therefore be considered exploratory.

## 3. Results

Initially, 102 patients were included in this study. However, only 96 patients completed the follow-up period and were included in the final analyses. Their clinical, functional, and radiological data were obtained in full. Six patients who did not complete the follow-up were excluded from the analyses, owing to non-clinical factors such as relocation, inability to establish contact, and logistical reasons. The baseline demographic and clinical characteristics of the cohort included in the analyses are presented in Table 1. The mean age was 58.0 ± 13.1 years, and 59.4% of patients were female. The most commonly affected spinal level was L4–5 (50.0%), followed by L5–S1 (19.8%) and L3–4 (21.9%). The preoperative mean VAS score was 8.02 ± 0.93.

### 3.1. Pain Outcomes

**Figure 2:**
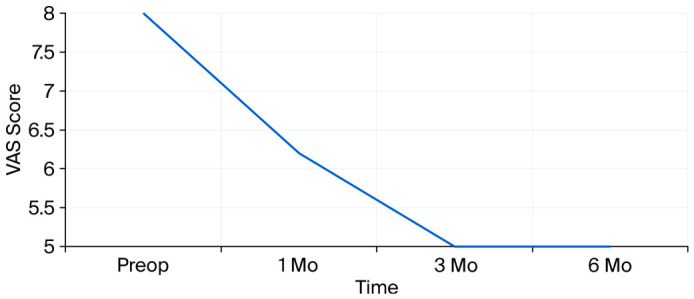
The change in VAS scores over time. Legend: Figure 2 illustrates the longitudinal change in the mean visual analogue scale (VAS) scores from baseline to 1, 3, and 6 months following PLDD. A progressive reduction in VAS scores is observed over time, indicating an improvement in pain intensity. The magnitude of change is consistent with clinically meaningful pain reduction across follow-up periods. Error bars represent standard deviation. Abbreviations: VAS = visual analogue scale; PLDD = percutaneous laser disc decompression; SD = standard deviation; Mo = month.

A significant and clinically meaningful decrease in VAS scores was observed after PLDD. The mean VAS score decreased from 8.02 preoperatively to 6.16 at 1 month postoperatively, 5.00 at 3 months, and 5.02 ± 1.99 at 6 months (all *p* < 0.001, Cohen’s d = −1.42 to −1.60). This sustained decline demonstrates that PLDD was associated with effective pain control in both the early and mid-term periods. The trends in VAS changes over time are presented in Table 2 and Figure 2.

### 3.2. Functional Outcomes

**Figure 3:**
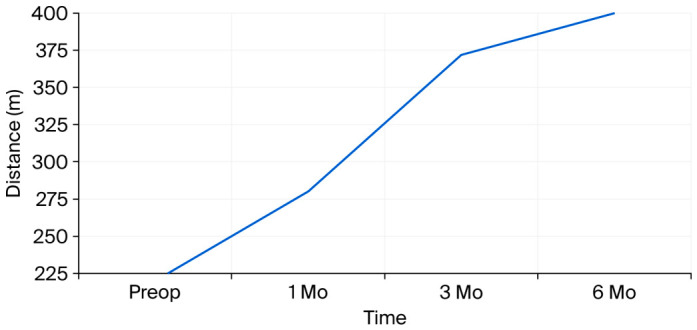
Pain-free walking distance over time. Legend: Mean pain-free walking distance at baseline and follow-up (1, 3, and 6 months). Increased distance indicates improvement in functional capacity. Abbreviations: m = metres; Mo = month.

Pain-free walking distance measurements, which assess functional capacity, showed statistically and clinically significant increases after treatment. The average walking distance increased from 212.7 m preoperatively to 284.9, 336.9, and 345.8 m at 1, 3, and 6 months, respectively (all *p* < 0.001, Cohen’s d = 0.45–0.78). This increase, exceeding 100 m in total, indicates that PLDD was associated with improvements in functional capacity. The details of these results are presented in Table 3 and Figure 3.

**Figure 4:**
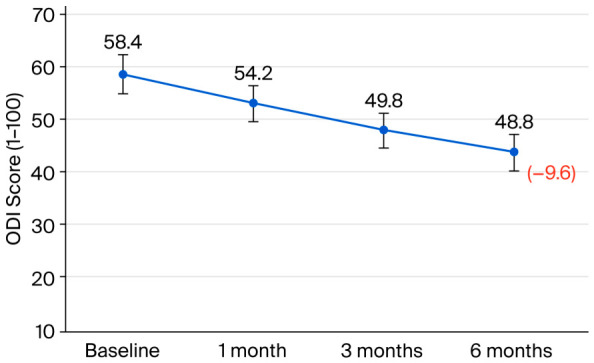
The change in the Oswestry Disability Index (ODI) over time. Legend: Figure 4 illustrates the longitudinal change in the mean Oswestry Disability Index (ODI) scores from baseline to 1, 3, and 6 months after PLDD. Although a progressive reduction in ODI scores is observed over time, the magnitude of improvement (−9.6 points at 6 months) remains below commonly accepted minimal clinically important difference (MCID) thresholds (12–15 points), thus indicating modest functional benefit. Notably, the limited reduction at 1 month suggests that early functional improvement may be minimal compared to pain reduction. Error bars represent standard deviation. Abbreviations: ODI = Oswestry Disability Index; PLDD = percutaneous laser disc decompression; SD = standard deviation; MCID = minimal clinically important difference.

The disability outcomes assessed using the ODI showed significant and progressive improvements following intradiscal laser decompression (PLDD). The mean ODI scores were 47.8 ± 10.3 in the preoperative period and decreased to 38.2 ± 10.9 at the 6-month follow-up, which indicates a statistically significant (*p* < 0.001) reduction in disability; however, the magnitude of this reduction (9.6 points) remains below commonly accepted MCID thresholds (12–15 points), suggesting that the functional benefit may be modest from a clinical perspective. Notably, the limited reduction observed at the 1-month follow-up (45.4) indicates that early functional improvement may be minimal. Detailed ODI changes over time are presented in Table 4 and Figure 4.

### 3.3. Radiological Findings

**Figure 5:**
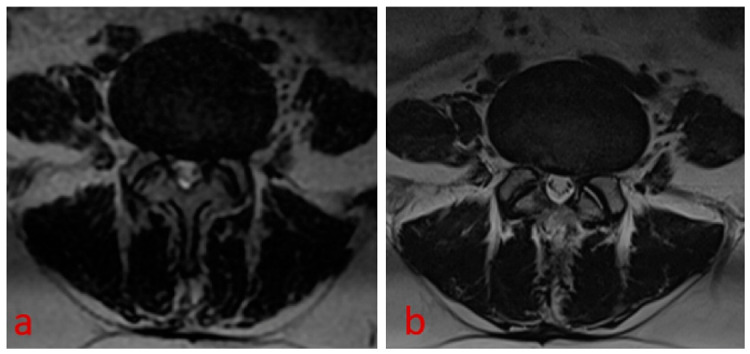
Representative axial lumbar MRI images of a patient undergoing percutaneous laser disc decompression. Legend: (**a**) Preoperative MRI showing lumbar disc protrusion associated with central canal narrowing and dural sac compression. (**b**) Three-month follow-up MRI after percutaneous laser disc decompression demonstrating partial reduction in disc protrusion and improved dural sac morphology. Radiological improvement should be interpreted cautiously because small changes in canal dimensions may be limited by measurement variability.

**Figure 6:**
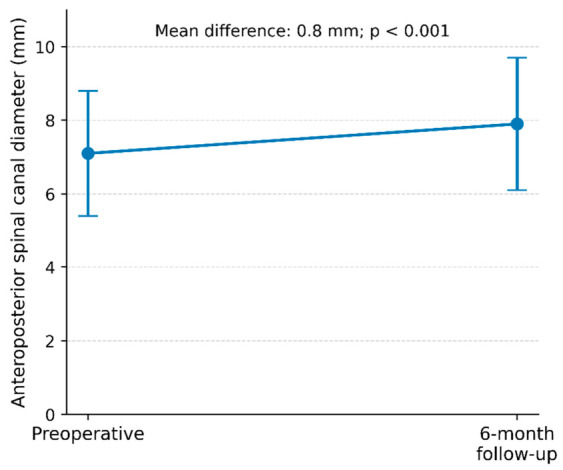
The change in spinal canal diameter. Legend: Figure 6 illustrates the change in the mean spinal canal diameter measured on MRI before and after PLDD. Although a statistically significant increase can be observed, the mean change (0.8 mm) is modest and may approach the limits of MRI measurement variability; therefore, its structural and clinical significance should be interpreted cautiously. Abbreviations: MRI = magnetic resonance imaging; mm = millimetres; PLDD = percutaneous laser disc decompression; SD = standard deviation.

Radiological evaluation demonstrated a significant increase in the anteroposterior diameter of the spinal canal, from 7.1 ± 1.7 mm preoperatively to 7.9 ± 1.8 mm at 3 months postoperatively (*p* < 0.001; Cohen’s d = 0.5), corresponding to a mean increase of 0.8 mm. Although statistically significant, this magnitude of canal enlargement was small and should not be overinterpreted as unequivocal evidence of clinically meaningful structural decompression (Figure 5). These radiological findings are summarized in Table 5 and Figure 6, and all reported values are consistent with the analyzed dataset.

#### Spinal Stenosis Subgroup Analysis

When the patients were analyzed according to their predominant stenosis patterns, the PLDD response exhibited phenotypic differences. The MCID response rates were 78%, 74%, and 61% in patients with lateral recess, foraminal, and mild to moderate central stenosis, respectively. A numerically higher response rate was observed in patients with lateral recess and foraminal stenosis; however, no formal statistical comparisons between subgroups were performed. These findings should be interpreted with caution and be considered exploratory and hypothesis-generating, rather than confirmatory.

### 3.4. MCID Response Analysis and Predictors

After 6 months, 72.9% of patients achieved a ≥2-point reduction in the VAS score, thus meeting the MCID criterion. Logistic regression analysis identified a higher preoperative VAS score (odds ratio [OR] = 1.38; *p* = 0.02) and an increase in canal diameter postoperatively (OR = 1.22; *p* = 0.03) as two key factors independently predicting MCID response within the limitations of the observational study design. In contrast, no significant effect was found for age, BMI, or comorbidities.

**Figure 7:**
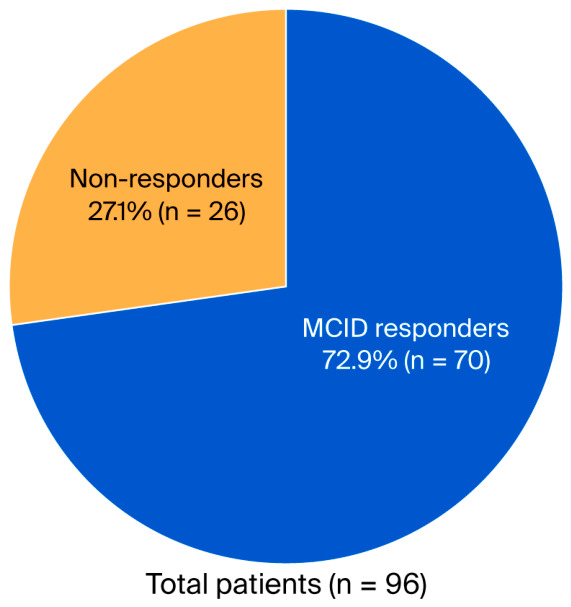
MCID responders at 6 months. Legend: The distribution of patients who achieved the minimal clinically important difference (MCID) at 6 months. A total of 72.9% of patients (70/96) met the MCID criterion, which was defined as a ≥2-point reduction in the VAS score, whereas 27.1% (26/96) were classified as non-responders. Abbreviations: MCID = minimal clinically important difference; VAS = visual analogue scale.

Five patients who did not achieve clinically meaningful improvement and were referred for open surgery were classified as non-responders in the MCID analysis, thus indicating that the MCID response rate in this study (72.9%) was calculated in a manner consistent with the clinical reality (Figure 7).

#### 3.4.1. Receiver Operating Characteristic (ROC) Analysis and Cut-Off Values

ROC curve analysis was performed to evaluate the predictive performance of variables associated with clinical response to PLDD. The area under the curve (AUC) was 0.7 for the preoperative VAS score and 0.8 for both the canal diameter increase and the combined model, thus indicating good predictive accuracy within this dataset. The optimal cut-off values were identified as a preoperative VAS score ≥ 7.5 and a canal diameter increase ≥ 0.3 mm. These thresholds may support clinical decision-making; however, external validation is required.

#### 3.4.2. Extended Multivariate Logistic Regression

Additional variables such as age, sex, BMI, affected level, disc type, comorbidities, and symptom duration were evaluated using an extended regression model. However, the preoperative VAS score (OR = 1.31; *p* = 0.02) and increased canal diameter (OR = 1.24; *p* = 0.01) remained two independent predictors. The lack of significance for other variables indicates that clinical response is more strongly associated with clinical severity and radiological response than demographic and anatomical factors, although residual confounding cannot be excluded due to the retrospective design of this study.

### 3.5. Safety Outcomes

PLDD is generally considered safe. Only two patients (2.1%) experienced short-term transient radicular irritation post-procedure, which resolved completely with conservative treatment. No major complications were observed, such as infection, hematoma, discitis, neurological deficits, or the need for re-intervention. The low complication rate supports the reliability of PLDD as a minimally invasive procedure within this cohort.

Moreover, during the follow-up period, five patients (5.2%) were referred for open lumbar surgery (microdiscectomy/decompression) because of an inadequate clinical response and persistent symptoms following PLDD. The average time to transition to surgery in these cases was calculated as 4.8 ± 1.6 months. No major neurological deficits developed in patients who underwent open surgery, and no complications were observed during postoperative follow-up; however, these findings should not be interpreted as comparative evidence against surgical treatment.

## 4. Discussion

To date, this study represents one of the largest single-centre Turkish cohorts evaluating PLDD performed using a standard energy protocol in patients with disc-related lumbar spinal stenosis. Our findings suggest that PLDD was associated with short-term improvement in pain and function, together with a modest increase in spinal canal diameter, in this cohort of carefully selected patients. These observations are broadly consistent with the existing literature, although they should be interpreted within the limitations of a retrospective single-centre design without a control group [13,14,15,16]. However, these observations are limited to the short-term to mid-term follow-up available in this study, and the persistence of benefit beyond 6 months cannot be determined from the present dataset.

### 4.1. Effect on Pain and Function

In our study, the significant reduction in VAS scores over 6 months was consistent with the results of numerous studies demonstrating the clinical efficacy of PLDD. A series of 71 patients from Turkey reported a marked reduction in pain and short-term treatment success after PLDD; similar results were observed in another series of 58 patients [17]. The significant increase observed in functional capacity (increase to 345.8 m) demonstrates that PLDD was associated with improvements in not only pain scores but also functional parameters reflected in daily living activities.

The significant decrease in ODI scores observed in this study suggests that PLDD not only reduces pain but also provides improvements in disability levels and daily functional capacity. However, the mean ODI reduction of 9.6 points remained below commonly accepted MCID thresholds (12–15 points), indicating that the observed disability improvement, although statistically significant, may be modest from a clinical standpoint. Additionally, the minimal change observed at the 1-month follow-up suggests that early functional recovery may be limited compared to pain reduction.

The continued decline in ODI values over time suggests that functional improvement may persist during the observed follow-up period, although the magnitude of change remained below usual MCID thresholds. Cohort data with 6- and 12-month follow-ups reporting improvements in ODI scores also support this finding [18].

Although the present data support early and mid-term improvement up to 6 months, the durability of these clinical gains beyond the observed follow-up window remains uncertain. In particular, whether the improvements in pain, walking capacity, and disability are maintained, attenuated, or require repeat intervention over longer follow-up cannot be determined from this retrospective cohort.

Consistent with these findings, a series of 30 patients reported a marked improvement in mid-term functional recovery in patients with small/medium disc volumes [18], another series indicated that PLDD is particularly successful in containing protrusions [19], and a study of 121 patients reported an analgesic response exceeding that of conservative treatment [20], thereby reinforcing the clinical efficacy of PLDD **as** reported in previous observational and comparative studies. When evaluated together with these data, the VAS score reduction and increase in walking distance observed in our cohort of 96 patients support the association between PLDD and a clinical improvement in appropriately selected patients with lumbar disc herniation [13,14,18,20].

Furthermore, a high MCID response rate of 78% was observed in our subgroup analysis, particularly in patients with lateral recess stenosis, thus indicating a numerically higher response in this subgroup; however, no statistical comparison between subgroups was performed, and these findings should therefore be interpreted with caution. These results should be considered exploratory and hypothesis-generating rather than confirmatory. This result suggests that patient selection should be optimized not only based on clinical criteria but also according to the radiological stenosis pattern.

### 4.2. Radiological Findings and Clinical Correlation

The mean increase in canal diameter was only 0.8 mm, and although statistically significant, this magnitude of change remains small. It may therefore reflect only limited structural expansion and should not be overinterpreted as the principal driver of clinical improvement [20,21].

Although studies have indicated that radiological changes do not always parallel clinical improvement [15], the moderate level of statistical significance associated with the clinical–radiological correlation in our study (r ≈ 0.52) suggests that radiological changes may be associated with, but do not fully explain, the clinical response. The fact that the radiological evaluations were performed by two blinded observers is an important strength of this study’s methodology [22].

Furthermore, the fact that radiological expansion, despite having a modest value of 0.8 mm on average, showed a significant relationship with clinical improvement suggests that the mechanism of action of PLDD cannot be attributed solely to geometric or mechanical decompression, as it also works in conjunction with biomechanical–biological processes, such as intradiscal pressure reduction, micro-level relief of nerve root compression, and decreased neurophysiological sensitivity. These findings support a multifactorial mechanism of action rather than a purely mechanical effect. Consequently, even small radiological changes may affect the treatment outcomes, although their clinical relevance remains uncertain.

### 4.3. MCID Response and Treatment Predictors

The finding that 72.9% of patients met the MCID criterion at 6 months demonstrates that PLDD was associated with a clinically meaningful improvement in a substantial proportion of patients. Similar studies have confirmed reduction in VAS score but reported limited responder analyses [22,23]. In our logistic regression analysis, high preoperative pain levels and greater canal expansion were identified as independent predictors of MCID response within the constraints of the observational study design. These results are consistent with those of Hashemi et al., which indicated that PLDD is more effective in patients with significant initial pain and radiologically successful decompression [24].

In the ROC analysis performed for the first time in this study, the preoperative VAS score (AUC = 0.7), canal diameter increase (AUC = 0.8), and the combination of these two parameters (AUC = 0.8) showed good predictive accuracy within the dataset used. The optimal cut-off values obtained (preoperative VAS ≥ 7.5, canal diameter increase ≥ 0.3 mm) could provide clinicians with objective decision support criteria for patient selection processes for PLDD; however, these thresholds require external validation in independent cohorts.

Furthermore, the absence of significant findings for age, sex, BMI, level, and disc type in the expanded multivariate model indicated that the treatment response was more strongly associated with clinical and radiological characteristics than demographic characteristics, although residual confounding cannot be excluded.

### 4.4. Comparison of Conservative Treatment and Surgery

Although conservative treatment is recommended as the first step in many patients with lumbar disc herniation [10], the efficacy of PLDD in resistant cases is supported by an increasing number of studies in observational and comparative contexts. A study of 121 patients showed that the VAS score reduction rate with PLDD was nearly seven times higher than that with conservative treatment [20]; however, the present study was not designed to perform such comparative analyses.

Compared with surgery, PLDD is reported to offer lower invasiveness, shorter recovery time, and a lower complication profile. A 2-year follow-up of 63 patients comparing open surgery with PLDD found similar pain outcomes [19], thus supporting the idea that surgery is only necessary for patients with neurological deficits or severe functional loss. Randomized trials comparing microdiscectomy and conservative treatment have shown that surgery enables faster recovery; however, the difference disappears in the long term [25,26], but the present study does not allow for conclusions regarding equivalence or superiority between these treatment modalities.

Large network meta-analyses examining endoscopic techniques and minimally invasive surgeries emphasize that surgical treatments are generally superior to conservative treatments; however, PLDD has not yet been sufficiently evaluated in comparative studies [26].

This situation indicates that PLDD requires a broader, multicentre, randomized study to clarify its relative efficacy. Our findings should therefore be interpreted as observational and hypothesis-generating rather than confirmatory and do not establish PLDD as superior or equivalent to other treatment modalities.

Therefore, PLDD is increasingly recognized as a treatment step of growing importance, particularly in patients who are not suitable for surgery or prefer to avoid surgery, owing to its low-risk profile and meaningful clinical benefits as suggested by observational evidence. In this study, the conversion rate to open surgery was 5.2%. This rate is consistent with the conversion rates to surgery after PLDD reported in the literature [27]. This low surgical escalation rate may suggest a potential role of PLDD in delaying surgical intervention in selected patients, although this cannot be confirmed without controlled comparative data. Accordingly, one of the key unresolved questions is not only whether PLDD provides short-term symptom relief, but also how durable this effect remains over longer follow-up and how often delayed symptom recurrence or surgical conversion occurs.

### 4.5. Safety and Complication Profile

No major complications were observed in our study, and only two patients (2.1%) experienced short-term transient radicular irritation, which is consistent with the low complication rates reported in the literature [16]. Although long-term reoperation rates after PLDD may vary [18], the early course is generally complication-free, and the safety profile is quite high, especially with good patient selection within the limits of this cohort. This also underscores that procedural safety and early tolerability should be distinguished from the durability of symptom control, which requires longer clinical follow-up.

Rare but serious complications have been reported following PLDD, such as discitis, osteomyelitis, and permanent neurological deficits [28], indicating that this procedure must be performed using appropriate techniques and suitable energy parameters and under sterile conditions at experienced centres. Epstein’s critical review emphasized that the off-label use of PLDD and similar percutaneous laser procedures or their application by practitioners with insufficient experience could pose unnecessary risks [29]. The absence of major complications in our cohort of 96 patients is consistent with careful patient selection (single-level, containing herniation, and conservative treatment-resistant cases) and the procedures being performed by a single experienced interventional pain specialist, although rare adverse events cannot be excluded in larger populations.

### 4.6. Strengths and Limitations of This Study

The limitations of this study include its retrospective design, the absence of a control group, which precludes causal inference and comparative interpretation, and the single-centre setting, which may limit generalizability. Although both walking distance and ODI were used to assess functional outcomes, the follow-up period was limited to 6 months; therefore, the durability of pain relief, functional improvement, and avoidance of further intervention beyond this period remains uncertain. This limitation is particularly relevant for PLDD, as a short-term favourable response does not necessarily predict sustained medium- to long-term benefit. In addition, the radiological assessment was restricted to the anteroposterior diameter of the spinal canal, whereas other potentially relevant parameters, such as canal area, foraminal area, and disc volume, were not analyzed. Patient-centred outcomes such as psychosocial status, return to work, and broader health-related quality-of-life measures were also not included. Furthermore, subgroup and ROC analyses were performed within an exploratory framework and require external validation.

### 4.7. Clinical Implications

The findings of this study suggest that PLDD may represent a minimally invasive treatment option in carefully selected patients with single-level, predominantly disc-driven lumbar spinal stenosis, particularly when symptoms persist despite conservative treatment and when surgery is either not preferred or not yet indicated. In this cohort, PLDD was associated with improvements in pain, functional capacity, and disability, together with a low complication rate. These observations support the potential role of PLDD as an intermediate treatment option for patients who remain symptomatic despite conservative management and are either not suitable for surgery or prefer to avoid it. However, these implications should be interpreted cautiously, as the present study does not establish comparative effectiveness relative to surgical or non-surgical alternatives.

## 5. Conclusions

This study demonstrated that PLDD was associated with significant short-term improvements in pain, function, and disability in patients with spinal stenosis in which the disc component is prominent, with improvements in clinical outcomes correlating with increases in spinal canal diameter. However, this relationship does not imply a direct causal or purely mechanical effect. The observed radiological expansion was statistically significant but modest in magnitude, and its clinical meaning should therefore be interpreted cautiously. Disability improvement in particular should be interpreted cautiously, as the mean ODI reduction remained below commonly used MCID thresholds.

The higher MCID response rates obtained in the subgroup analysis of patients with predominant lateral recess and foraminal narrowing indicate a numerically higher response in these subgroups, and these findings should be interpreted as exploratory due to the absence of statistical comparisons and prespecified hypotheses. However, the retrospective design, lack of a control group, and 6-month follow-up period indicate that caution should be exercised when interpreting the findings. The threshold values of a preoperative VAS score of ≥ 7.5 and canal diameter increase of ≥ 0.3 mm defined in the ROC analysis may offer potentially useful decision support indicators for predicting treatment response, although these thresholds require external validation.

Conversion to open surgery was required in a small proportion of cases, thus demonstrating that PLDD is not a substitute for surgery. This study does not establish its equivalence or superiority relative to surgical treatment, but rather, it indicates that it is a preoperative intermediate treatment step for certain patient groups based on the observational findings.

Accordingly, PLDD should be viewed as a selectively applicable minimally invasive option rather than a general treatment for all forms of lumbar spinal stenosis. In appropriately selected patients with disc-related lumbar spinal stenosis, particularly when the stenotic narrowing is predominantly driven by disc pathology, PLDD may be considered a low-complication minimally invasive treatment option. However, the observed clinical benefit in this study was limited to a 6-month follow-up period, and its persistence beyond this timeframe remains uncertain. In this cohort, PLDD was associated with short-term improvements in pain and functional capacity; however, no conclusions can be drawn regarding its comparative effectiveness relative to other treatment modalities. Further well-designed prospective studies with longer follow-up are required to better define the long-term role of this method and to allow valid comparisons with alternative treatment strategies.

## Figures and Tables

**Table 1:** Baseline demographic and clinical characteristics.

Variable	Value
Total number of patients (n)	96
Age (years)	58.0 ± 13.1 (range 33–81)
Sex, n (%)	Male: 39 (40.6%); Female: 57 (59.4%)
Primary diagnosis, n (%)	Disc herniation: 72 (75%); Degeneration: 24 (25%)
Disc type, n (%)	Herniation: 67 (69.8%); Degeneration: 29 (30.2%)
Side, n (%)	Right: 53 (55.2%); Left: 43 (44.8%)
Level distribution, n (%)	L2–3: 8 (8.3%); L3–4: 21 (21.9%); L4–5: 48 (50.0%); L5–S1: 19 (19.8%)
Preoperative VAS score	8.02 ± 0.93
Preoperative walking distance (m)	212.7 ± 148.3
Subsequent open surgery during follow-up, n (%)	5 (5.2%)

Legend: Baseline demographic and clinical characteristics. Continuous variables are presented as means ± SD with ranges. Categorical variables are presented as numbers and percentages. Abbreviations: VAS = visual analogue scale; SD = standard deviation; m = metres.

**Table 2:** VAS change.

Time	Mean ± SD	*p*-Value	Cohen’s d
1 Month	6.16 ± 1.61	<0.001	−1.496
3 Months	5.00 ± 2.04	<0.001	−1.602
6 Months	5.02 ± 1.99	<0.001	−1.630

Legend: Comparison of pre- and postoperative VAS scores at each follow-up period. Statistical significance was assessed using repeated-measures analysis with post hoc comparisons. Cohen’s d indicates effect size, and negative values represent pain reduction. Abbreviations: VAS = visual analogue scale; SD = standard deviation; d = Cohen’s d.

**Table 3:** Change in walking distance.

**Time**	**Mean ± SD (m)**	***p*** **-Value**	**Cohen’s d**
1 month	284.9 ± 200.4	<0.001	0.458
3 months	336.9 ± 236.6	<0.001	0.740
6 months	345.8 ± 237.9	<0.001	0.822

Legend: Changes in pain-free walking distance during follow-up. Statistical significance was assessed using repeated-measures analysis with post hoc comparisons. Cohen’s d reflects functional improvement. Abbreviations: m = metres; SD = standard deviation; d = Cohen’s d.

**Table 4:** Change in Oswestry Disability Index (ODI) over time.

Time Point	ODI (Mean ± SD)
Preoperative	47.8 ± 10.3
1 month	45.4 ± 11.0
3 months	41.8 ± 10.9
6 months	38.2 ± 10.9

Overall *p*-value: <0.001. Mean change (baseline to 6 months): −9.6 points. Interpretation: Below the commonly accepted MCID threshold (12–15 points). Legend: Table 4 shows the longitudinal changes in ODI scores from baseline to 1, 3, and 6 months following PLDD. Although statistically significant, the magnitude of improvement remains below MCID thresholds, indicating modest functional benefit. Early changes suggest limited short-term functional recovery. Abbreviations: ODI = Oswestry Disability Index; PLDD = percutaneous laser disc decompression; SD = standard deviation; MCID = minimal clinically important difference.

**Table 5:** Spinal canal diameter.

Parameter	Mean ± SD (mm)	*p*-Value	Cohen’s d
Preoperative	7.1 ± 1.7	-	-
Postoperative	7.9 ± 1.8	<0.001	0.5

Legend: Comparison of preoperative and postoperative spinal canal diameters measured using MRI. Statistical significance was assessed using repeated-measures analysis. Cohen’s d was calculated to determine magnitude of radiological improvement. Abbreviations: mm = millimetres; SD = standard deviation; d = Cohen’s d.

## Data Availability

The datasets used and/or analyzed during the current study are available from the corresponding author upon reasonable request.
